# Cell Penetrating Peptide Conjugated Chitosan for Enhanced Delivery of Nucleic Acid

**DOI:** 10.3390/ijms161226142

**Published:** 2015-12-04

**Authors:** Buddhadev Layek, Lindsey Lipp, Jagdish Singh

**Affiliations:** 1 Department of Pharmaceutics, College of Pharmacy, University of Minnesota, 308 Harvard Street S.E., Minneapolis, MN 55455, USA; 2 Department of Pharmaceutical Sciences, School of Pharmacy, North Dakota State University, Fargo, ND 58105, USA; lindsey.will@ndsu.edu

**Keywords:** chitosan, gene delivery, cell penetrating peptides (CPPs), cellular uptake, nonviral vector, transfection

## Abstract

Gene therapy is an emerging therapeutic strategy for the cure or treatment of a spectrum of genetic disorders. Nevertheless, advances in gene therapy are immensely reliant upon design of an efficient gene carrier that can deliver genetic cargoes into the desired cell populations. Among various nonviral gene delivery systems, chitosan-based carriers have gained increasing attention because of their high cationic charge density, excellent biocompatibility, nearly nonexistent cytotoxicity, negligible immune response, and ideal ability to undergo chemical conjugation. However, a major shortcoming of chitosan-based carriers is their poor cellular uptake, leading to inadequate transfection efficiency. The intrinsic feature of cell penetrating peptides (CPPs) for transporting diverse cargoes into multiple cell and tissue types in a safe manner suggests that they can be conjugated to chitosan for improving its transfection efficiency. In this review, we briefly discuss CPPs and their classification, and also the major mechanisms contributing to the cellular uptake of CPPs and cargo conjugates. We also discuss immense improvements for the delivery of nucleic acids using CPP-conjugated chitosan-based carriers with special emphasis on plasmid DNA and small interfering RNA.

## 1. Introduction

Gene therapy is the transfer of exogenous genetic materials to replace a faulty gene or introduce a new gene into the body in an attempt to treat genetic diseases or enhance the body’s ability to fight a disease. The concept of gene therapy was first described in 1972 by Friedmann and Roblin who strongly urged caution before initiating human gene therapy studies [1]. Initially, gene transfer was considered an experimental technique to understand gene function and its regulation. However, recent advances in molecular biology and biotechnology, and completion of the Human Genome Project have accelerated the detection of numerous disease-causing genes. Therefore, it is not hard to envision treatment of genetic disorders such as cystic fibrosis [2], hemophilia [3], muscular dystrophy [4], or X-linked severe combined immunodeficiency [5]. The scope of gene therapy also encompasses numerous acquired diseases, such as cancers [6,7,8], cardiovascular diseases [9,10], neurodegenerative diseases [11,12,13], and infectious diseases [14,15,16]. By July 2015, over 2210 gene therapy clinical trials had been conducted or approved in 36 countries. Consequently, gene therapy has emerged as an alternative treatment modality against many human diseases.

However, the delivery of unprotected genes is unrealistic due to their susceptibility toward nuclease degradation, rapid clearance by macrophages, and non-specificity to target cells [17]. Furthermore, the large size, high anionic charge density, and hydrophilic nature of the nucleic acids impose a significant barrier to their intracellular delivery and overall gene transfection efficiency [18]. Therefore, the primary challenge for successful gene therapy involves finding the best approach to deliver functional genes into the target cells of the patient. Scientists have pursued a number of gene delivery systems, which could be broadly categorized as viral and nonviral systems.

Although most of the ongoing or approved gene therapy protocols use viral vectors for gene delivery, there is increasing interest in developing nonviral vectors to circumvent viral associated safety issues such as potential infectivity, immunogenicity, inflammation, and insertional mutagenesis [19,20,21,22]. Nonviral gene delivery systems also offer inherent formulation flexibility and cost-effective large scale manufacturing [23]. Most of the nonviral vectors fall into two main categories: (i) cationic liposomes (lipids) such as [1,2-bis(oleoyloxy)-3-(trimethylammonio)propane] (DOTAP) [24], *N*-[1-(2,3-dioleyloxy)propyl]-*N*,*N*,*N* trimethylammonium chloride (DOTMA) [25], and 3β[*N*-(*N*′,*N*′-dimethylaminoethane)-carbamoyl]cholesterol (DC-Chol) [26]; and (ii) cationic polymers such as polyethyleneimine [27], poly-l-lysine [28], polyamidoamine [29], chitosan [30], and cell penetrating peptides (CPPs) [31]. Recently, chitosan-based delivery systems have received increasing attention as a safe carrier for genetic material including, plasmid DNA (pDNA) and small interfering RNA (siRNA).

Chitosan is a straight chain natural polysaccharide composed of randomly distributed β-(1→4)-linked d-glucosamine and *N*-acetyl-d-glucosamine [32]. Commercial chitosan is primarily prepared by alkaline *N*-deacetylation of chitin, normally found in the exoskeleton of crustaceans (crabs, shrimps, cuttlefish, and lobsters) and cell walls of fungi [32,33,34]. The d-glucosamine unit has a p*K*a value of ~6.5, which leads to the protonation of free amine groups in acidic to neutral pH [35,36]. Chitosan displays several beneficial qualities such as excellent biocompatibility, negligible immunogenicity, and low cytotoxicity, which make it an attractive polymer for pharmaceutical applications [37,38]. Due to its cationic charge, chitosan can form nanoscale complexes (polyplexes) with negatively charged nucleic acids through electrostatic interactions and thereby provide efficient protection to complexed nucleic acids. Despite the huge potential of chitosan as a gene carrier, its poor transfection efficiency has greatly impeded its clinical applications.

Over the years, numerous chemical modifications of chitosan have been pursued to enhance its gene transfection efficiency. Most commonly, chitosan was structurally modified with hydrophobic [39,40,41,42,43,44,45,46], hydrophilic [47,48], amphiphilic [49,50], CPPs [46,51], and cell specific ligands [52,53,54] using the reactive amino group (C2 position) and hydroxyl groups (C6 and C3 positions). The purpose of this review is to discuss the role of CPPs in increasing the transfection efficacy of chitosan and the recent development in the CPP-conjugated chitosans as nucleic acid carriers.

## 2. Cell Penetrating Peptides (CPPs) and Their Classification

CPPs are also called “Trojan Horse” peptides, protein transduction domains (PTDs), or membrane-permeable peptides (MPPs). CPPs are a class of diverse peptides, typically consisting of 5–30 amino acids, which are distinguished by their ability to translocate though various biological membranes. The first CPP was discovered independently by two different research groups in 1988, when it was noticed that the trans-activating transcriptional activator (TAT) protein of HIV-1 could translocate the cell membrane and enter the nucleus [55,56]. After a few years, the third helix of Drosophila Antennapedia homeodomain protein was shown to be taken up by the cells in culture [57,58]. These discoveries were followed by the identification of the minimal TAT peptide sequence (47YGRKKRRQRRR57) essential for cellular entry [59]. Since then, several other proteins and peptides capable of cell membrane translocation have been discovered, and the list of the CPPs has been extended rapidly. These include VP22, model amphipathic peptide (MAP), transportan, Pep-1, synthetic polyarginines, and signal sequence-based peptides [60,61,62].

**Figure 1:**
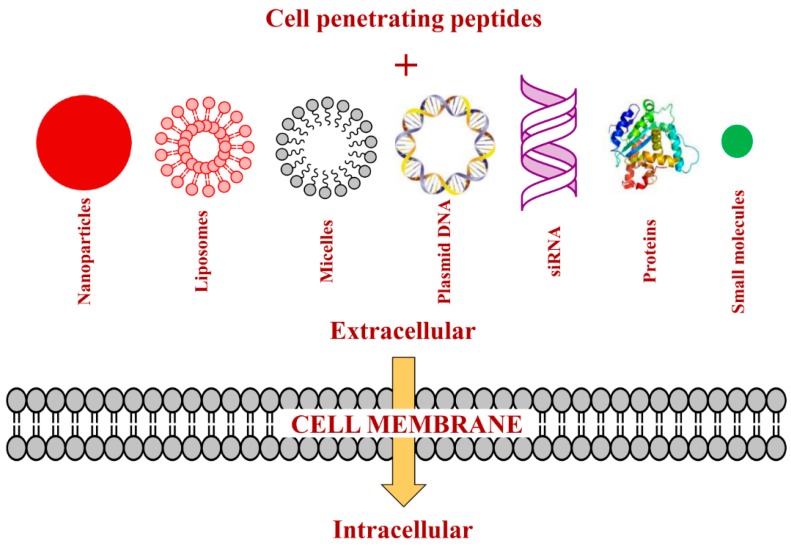
Applications of cell penetrating peptides (CPPs) for intracellular delivery of diverse cargoes.

Over the past few years, there has been an increased application of CPPs for the delivery of diverse therapeutic cargoes such as proteins, peptides, nucleic acids, liposomes, nanoparticles, micelles, and small molecule drugs across the cell membrane (Figure 1) [51,60,63,64,65]. CPP-mediated transport offers several potential benefits over traditional techniques, including low toxicity, dose-dependent efficiency, efficiency for a variety of cell types, and flexibility regarding the size or type of cargo [66]. These intrinsic features of CPPs have encouraged their applications in designing novel therapeutics against numerous diseases, including heart disease, stroke, cancer, and pain [61]. Currently, several preclinical and clinical trials are ongoing that utilize CPPs as a therapeutic carrier, such as AZX100 (for keloid scarring, Phase II, Capstone Therapeutics, Tempe, AZ, USA), KAI-9803 (for myocardial infarction, Phase II, KAI Pharmaceuticals, South San Francisco, CA, USA), RT001 (for wrinkling skin, Phase II, ReVance Therapeutics, Newark, NJ, USA), XG-102 (for hearing loss, Phase II, Auris Medical, Basel, Switzerland), and DTS-108 (for cancer, Preclinical, Diatos SA, Paris, France) [67]. Besides therapeutic applications, CPPs have also been utilized for transport of fluorescent or radioactive agents for imaging purposes [68].

The significant heterogeneity among various CPPs makes a clear and precise classification very difficult. CPPs can be classified into several subgroups based on their origin or physicochemical characteristics [67,68,69,70,71,72]. According to their origin, CPPs can be assigned to three main classes: protein-derived CPPs, chimeric CPPs, and synthetic CPPs [67,68,73,74]. Protein-derived CPPs, also called PTDs, are short sequences of the protein domain essential for translocation ability, examples are penetratin and TAT. Chimeric CPPs are a fusion of two or more natural peptide sequences, for instance transportan, which is a chimeric peptide of galanin and mastoparan. Synthetic CPPs are developed using rational design, prediction programs, or even via trial and error method. Members of this class include polyarginines, MAP, GALA, and KALA.

Depending on physicochemical characteristics, CPPs are again classified into three main categories: cationic CPPs, amphipathic CPPs, and hydrophobic CPPs [69,70,71,72]. Cationic CPPs, also designated as low amphipathic peptide, are comprised of clusters of cationic amino acids such as arginine or lysine in their sequence. The examples of cationic CPPs include TAT, penetratin, P22N, octaarginine (R8), *etc*. Cellular uptake potential of cationic CPPs strongly depends on the occurrence of arginine units, which can interact with sulfated proteoglycans and anionic phospholipids of cell membranes via electrostatic interactions. Amphipathic CPPs contain sequential hydrophobic and hydrophilic domains which are crucial for their translocation efficiency. This class includes transportan, MAP, CADY, *etc*. The hydrophobic CPPs hold a low net charge (>20% of the sequence) and primarily consist of nonpolar amino acids. Examples of this class include MPG peptides, peptide of Kaposi fibroblast growth factor (K-FGF), C105Y, and vascular endothelial-cadherin (pVEC).

## 3. Strategies of Attaching CPPs to Cargos

Two different CPP-mediated transport strategies have been reported to date; the CPP is either covalently conjugated to the cargo or forming a non-covalent stable complex with its cargo. Proteins and peptides are linked to CPPs through a disulfide bond after modifying CPP and protein/peptide with cysteine or using a cross-linker [75]. Similarly, nucleic acids can be attached to CPP through a cleavable disulfide, amide, or hydrazine bonds. For example, siRNAs have been covalently linked to penetratin [76,77] and transportan [77] via disulfide bond to enhance their intracellular delivery. In another study, a siRNA-Tat conjugate was prepared via stable thiomaleimide linkage for enhanced EGFP gene silencing in HeLa cells [78]. Although covalent strategies have been successfully utilized for delivering a wide range of cargoes, this method is restricted from the chemical point of view, as there is a risk of altering the biological activity of the cargo [79]. The non-covalent strategy depends on the amphipathic properties of short peptides such as MPG, Pep-1, or CADY that can form stable complexes with cargoes without requiring any chemical modification or crosslinking. MPG shows strong affinity for charged molecules, while Pep-1 is more suitable for proteins/peptides and neutral DNA mimics [80,81]. CADY is less selective for its cargo and can form stable complexes with both charged and neutral molecules [82,83]. These peptides display a strong affinity for their respective cargoes such as proteins/peptides or oligonucleotides even at nanomolar concentration and form stable complexes through electrostatic and hydrophobic interactions [82,84]. Other CPPs have been explored recently for their non-covalent complex formation capabilities with oligonucleotides and proteins. These CPPs include TAT [79,85,86], penetratin [87], poly-arginine [88], and transportan [89]. The non-covalent strategy offers several potential advantages over covalent counterpart, including no need for chemical cleavage which facilitates the efficient release of the cargo and allow modifications to increase specificity for the cargo or the target [90].

## 4. Mechanisms of Cellular Uptake of CPPs and Their Conjugates

**Figure 2:**
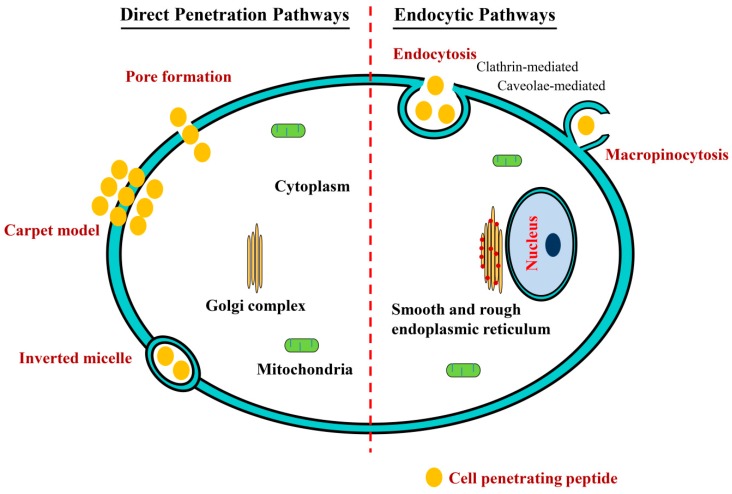
Cellular uptake mechanisms of CPPs and their conjugates with direct pathways illustrated to the left of the red dashed line and endocytic pathways to the right.

A proper recognition of the mechanism of uptake and intracellular tracking of nucleic acid carriers is a key step of optimization for maximum efficacy. Although CPPs have been extensively used to deliver numerous cargo molecules into cells, the exact pathways of cellular internalization have yet to be resolved [91,92]. Also, most CPP molecules can be internalized by cells via two or more pathways based on the characteristic of the CPP/cell interaction. Here, we briefly discuss the two major cellular uptake mechanisms involved in the internalization of the free or cargo-conjugated CPPs which comprise direct penetration (*i.e.*, energy-independent or non-endocytic pathways) and endocytic pathways (Figure 2).

### 4.1. Direct Penetration Pathways

The direct translocation of CPPs via energy-independent pathways includes inverted micelle model [93], pore formation [94], and carpet-like model [95]. The first step in direct translocation involves electrostatic interactions between cationic CPPs and anionic constituents of the cell membranes such as sulfated proteoglycans and phospholipids. In an inverted micelle model, the cationic residues of CPPs interact with the negatively charged phospholipids of cell membranes, which leads to interaction of hydrophobic residues with the membrane core followed by destabilization of the cell membrane and formation of an invaginated membrane [96]. The simultaneous reorganization in adjacent lipids results in inverted micelle formation that encloses CPPs in its hydrophilic core [97]. The consequential membrane disruption releases CPPs into the intracellular compartment. This model has been observed with CPPs like penetratin in which tryptophan residues hydrophobically interact with the cell membrane [98]. However, this model is not adequate to explain the uptake of highly cationic CPPs such as polyarginine or TAT peptides, which are devoid of hydrophobic amino acids [66]. Moreover, this model is not feasible with the uptake of high molecular weight cargoes, since formation of inverted micelles with a hydrophilic core that can encapsulate large size cargoes is unlikely to occur. According to the pore formation models, including both barrel-stave and toroidal pore model, the cellular internalization of CPPs and their conjugates would result from the creation of transient pores in the cell membrane. In the case of the barrel-stave model, the pores appear when outwardly facing hydrophobic residues of α-helical CPPs interact with aliphatic chains of phospholipids and the inwardly facing hydrophilic residues form the pore [99,100]. The basic mechanism is also similar in the case of toroidal pore model, except the CPPs interact exclusively with the hydrophilic groups of the membrane phospholipids, inducing significant rearrangement of the lipid membrane [99,101]. Primary amphipathic peptides such as Pep-1, transportan, and MPG translocate through pore formation [102]. The pores appear when concentration of these peptides crosses the threshold value, which varies among different peptides [103]. Based on the carpet model, the cellular uptake of CPPs and their conjugates occur as a result of a transient disruption of the cell membrane caused by the extensive interactions of the hydrophobic residues of CPPs and the membrane's hydrophobic core and subsequent phospholipid rearrangement [99].

### 4.2. Endocytosis

Classifications of endocytosis fall within two major categories, namely phagocytosis and pinocytosis. Phagocytosis is primarily observed in specialized cells such as macrophages, monocytes, and neutrophils that function as a major mechanism to clear pathogens and cell debris. Pinocytosis comprises macropinocytosis, clathrin-or caveolae-mediated endocytosis, and clathrin-or caveolae-independent endocytosis [104,105]. Uptake of large cargos (>1 µm) conjugated to CPPs tend to be mediated via macropinocytosis, which involves an inward folding of the cell membrane's outer surface to form a vesicle around the CPP-cargo conjugates called macropinosome. Subsequently, the macropinosome is transported within the cytoplasm and fuses with endosomes and lysosomes [106]. Receptor mediated endocytosis involves a clathrin-or caveolin-dependent uptake mechanism. Clathrin and caveolin proteins are required for membrane invagination and help in vesicle formation after binding of CPP-cargo conjugate to the membrane receptor [104]. Clathrin-coated vesicles are found to be about several hundred nanometers in diameter, while caveolin-pits are about 50–80 nm in diameter [104,105].

Regarding cargo-conjugated CPPs, most of the recent studies indicate that endocytosis strongly contributes the cellular uptake of CPP-cargo conjugates. However, there is still some controversy about the exact endocytic mechanism involved in the uptake process. The cellular uptake of CPP-cargo conjugates could be significantly influenced by several factors, including distinctive features of the cargo (size, charge, and type), physicochemical properties of CPPs (molecule length, charge, and hydrophobicity), experimental conditions (CPPs concentration, incubation time, temperature, and buffer), and cell properties (membrane composition, size, state, and also number of passages) [107,108,109]. For example, TAT has been shown to internalize through lipid-raft-dependent endocytosis, caveolae-mediated endocytosis, and macropinocytosis when the cargoes were large (proteins and quantum dots), and via clathrin-mediated endocytosis after conjugation with a fluorophore [110]. In a separate study, Lundin *et al.* [111] compared the cellular uptake mechanism of highly cationic CPPs (TAT, M918, and penetratin) and amphiphilic CPPs (MAP, transportan, TP10, pVEC) when conjugated with peptide nucleic acids. The results revealed that cationic conjugates were mainly internalized through macropinocytosis, while amphipathic peptide conjugates relied on clathrin-mediated endocytosis. Moreover, the concentrations of CPPs can also influence the mode of uptake mechanism. For example, both endocytic and direct translocation pathways were involved in the uptake of TAT at high concentrations (>10 μM), while caveolae/lipid-raft-mediated endocytosis and macropinocytosis were predominant below 10 μM concentration [112]. Examples of a few commonly used CPPs with their origin, and uptake mechanisms are summarized in Table 1.

**Table 1:** Examples of common cell penetrating peptides (CPPs), their origins, sequences, and cellular uptake mechanisms.

Name	Origin	Sequence	Cellular Uptake Mechanism	Reference
**TAT**	Protein-derived from HIV-1 TAT protein	GRKKRRQRRRPPQ	Macropinocytosis, endocytosis, direct penetration	[69,72]
**Penetratin**	Protein-derived form Drosophila antennapedia	RQIKIWFQNRRMKWKK	Macropinocytosis, endocytosis, direct penetration	[69,71,72,113]
**Transportan**	Chimeric peptide of galanin and mastoparan	GWTLNSAGYLLGKINLKALAALAKKIL	Endocytosis, direct penetration	[72]
**MAP**	Synthetic peptide	KLALKLALKALKAALKLA	Energy dependent and energy independent endocytosis	[114,115]
**VP22**	Protein-derived from HSV-1	DAATATRGRSAASRPTERPRAPARSASRPRRPVD	Endocytosis	[116]
**KALA**	Synthetic peptide	WEAKLAKALAKALAKHLAKALAKALKACEA	Endocytosis	[69]
**GALA**	Synthetic peptide	WEAALAEALAAEALAEHLAEALAEALEALAA	Endocytosis	[69]
**Pep-1**	Chimeric: HIV-reverse transcriptase/SV40 T-antigen	KETWWETWWTEWSQPKKKRKV	Direct penetration	[72]
**MPG**	Chimeric: HIV-gp41/SV40 T-antigen	GALFLGFLGAAGSTMGAWSQPKKKRKV	Direct penetration	[108]
**Polyarginines**	Synthetic peptide	Rn (6 < *n* < 12)	Macropinocytosis, endocytosis, direct penetration	[72]
**CADY**	Synthetic peptide	GLWRALWRLLRSLWRLLWRA	Direct penetration	[117]

## 5. Recent Progress in CPP Conjugated Chitosan

Gene therapy is an emerging therapeutic strategy for the treatment of a wide range of genetic disorders. The concept of gene therapy involves nucleic acid (pDNA, antisense oligonucleotides, microRNA, or small hairpin RNA) delivery to the intracellular compartment to modulate gene expression in target cell populations and thereby control cellular functions and responses. However, systemic delivery for unprotected nucleic acids is restricted by their susceptibility to endonuclease degradation, expeditious renal clearance, off-target distribution, and poor cellular uptake [17,18]. Therefore, the success of nucleic acid-based therapeutics is largely dependent on the development of safe and efficient vectors which allow their delivery to the suitable cellular compartment of target cells.

A diverse range of materials have been explored to address key challenges of *in vivo* nucleic acid delivery, which includes cationic lipids [25,26,118], peptides [31,119], cationic polymers [27,29,30,120], aptamers [121], and antibodies [122,123]. Among them, polymeric vectors have received increasing interest for nucleic acid delivery since they could be chemically modified to ensure high intracellular deposition. Recently, chitosan-based carriers have gained increasing attention due to their high cationic charge density, excellent biocompatibility, low cytotoxicity, negligible immunogenicity, and ease of chemical conjugation. However, one of the major shortcomings of chitosan as a DNA or siRNA delivery vector is its inefficient cellular uptake [37,38]. Conjugation of CPPs to chitosan is therefore viewed as a promising approach to improve cellular uptake of chitosan based formulations. Furthermore, CPPs also facilitate endosomal escape of its cargo, which ultimately enhance the efficacy of the nucleic acid-based formulations [124]. Recent advancements in CPP conjugated chitosan-based nucleic acid delivery systems are presented in Table 2.

### 5.1. CPP Conjugated Chitosan for DNA Delivery

To develop chitosan-based gene carriers capable of inducing efficient gene expression, chitosan is often modified with various CPPs, which include TAT, penetratin, and octaarginine. Thiolated chitosan possess enhanced mucoadhesiveness and cell permeation properties which could lead to increased gene transfection ability *in vitro* and likewise *in vivo* [125]. Additionally, the disulfide bonds within the thiomers might undergo a thioldisulfide exchange reaction in association with cytoplasmic glutathione, triggering intracellular release of pDNA [126]. Additionally, chemical conjugation of CPP to nanoparticulate delivery systems is a promising strategy to improve cellular uptake of pDNA. Therefore, the coupling of CPP to thiolated chitosan/pDNA polyplexes could offer the benefits of both systems for improved transfection efficiency. With the aim of developing an efficient gene delivery system, Rahmat *et al.* [46] has prepared nanoparticles by complex coacervation of pDNA and chitosan-thioglycolic acid (TGA) polymer and chitosan-TAT peptide polymer. Incorporation of TAT to chitosan-TGA/pDNA nanoparticles stimulated cellular uptake and endosomal escape of nanoparticles. Consequently, the nanoparticles prepared by combining chitosan-TAT peptide and chitosan-TGA have shown 7.12- and 67.37-fold higher gene transfection in comparison to unmodified chitosan and naked pDNA, respectively.

**Table 2:** Representative examples of CPP-conjugated chitosan used in DNA/siRNA delivery.

CPP	Chitosan Complex	Nucleic Acid	Cells/Model	Effect	Reference
Poly-l-arginine (PLR)	PEGylated PLR-grafted chitosan (PEG-CS-PLR)	siSVN, siGFP, siRFP	Hepa 1–6, A549, VK2 cells, and 293 T-GFP cells	Increased serum stability and reduced cytotoxicity Increased cellular delivery efficiency of siRNA Silenced abnormally overexpressed genes in tumor tissues *in vivo*	[127]
			Mice bearing B16F10-RFP tumors		
Octaarginine	Octaarginine-modified chitosan (R_8_-CS)	pGL3	COS-1 cells	Increased serum stability and reduced cytotoxicity Enhanced gene transfection	[128]
TAT	Chitosan-thioglycolic acid (CS-TGA) + Chitosan-TAT (CS-TAT)	pEGFP	HEK293 cells	Improved cellular uptake and endosomal escape Enhanced gene transfection	[46]
Nonaarginine	Nonaarginine-modified chitosan (R_9_-chitosan)	siCypB	HeLa cells	Improved siRNA binding affinity and cellular uptake Increased gene silencing	[129]
TAT	TAT peptide-tagged PEGylated chitosan (CS-PEG-TAT)	siGLO	Neuro2a cells	Improved siRNA binding affinity and stability of the polyplex Enhanced transfection efficiency	[130]
Penetratin	Linoleic acid and penetratin dual-functionalized chitosan (CS-Lin-Pen)	pGFP, pβ-gal	HEK293, CHO, and HeLa	Improved cellular uptake and nuclear localization Enhanced gene transfection	[51]
TAT	TAT-LHRH-chitosan conjugate (TLC)	pGL3	BEL-7402, L02 cells	Increased DNA condensing ability Promising transgenic efficacy and high selectivity for hepatoma cells both *in vitro* and *in vivo* Decreased cytotoxicity	[131]
TAT	TAT tagged and folate modified *N*-succinyl-chitosan (TAT-Suc-FA)	pDNA	K562 cells	Increased DNA condensing ability Decreased cytotoxicity	[132]

To improve the transfection effectiveness and specificity, a bifunctional peptide of TAT along with luteinizing hormone-releasing hormone (LHRH) was chemically conjugated to low molecular weight chitosan, to create a TAT-LHRH-chitosan conjugate (TLC) [131]. The TLC showed stronger pDNA condensing capacity compared to unmodified chitosan and formed stable nanoscale TLC/pDNA polyplexes (70–85 nm) with a net positive surface charge of approximately +30 mV. The expanded stability of TLC/pDNA polyplexes was attributed to the raised isoelectric point of TLC (11.3) compared to unmodified chitosan (6.3), which confers TLC with a higher positive-charge density and allows the construction of polyplexes with pDNA that are more stable at physiological pH. As a result, conjugation of TAT peptide conveys immense influence to increase the charge density of TLC, as it consists of eight cationic amino acid molecules. On the other hand, polyplexes formed with low molecular weight chitosan and pDNA were unstable and non-uniform in size. Notably, the TLC/pDNA polyplexes delivered pDNA at a 14-fold higher amount into hepatoma cells (BEL-7402) rather than healthy liver cells (L02), which led to 110-fold greater gene transfection in BEL-7402 as compared to L02 cells. Just as noteworthy, the transfection efficiency of TCL/pDNA polyplexes in BEL-7402 cells was around 20 times greater than the commercial transfection agent Lipofectamine 2000.

In a very recent study, Yan *et al.* [132] synthesized a TAT and folic acid dual functionalized *N*-succinyl-chitosan conjugate (TAT-Suc-FA) as a potential tumor targeted gene carrier. The electrostatic interaction between TAT-Suc-FA copolymer and pDNA led to the spontaneous formation of stable cationic polyplexes with diameters between 54 to 100 nm. Moreover, TAT-Suc-FA copolymer was less cytotoxic than unmodified chitosan within a specified concentration range (2–500 µg/mL). These results demonstrate how TAT-Suc-FA could act as a future gene delivery vector.

With the desire of tethering the individual benefits of cationic micelles and CPP, a novel chitosan-based polymer dual functionalized with linoleic acid and penetratin (CS-Lin-Pen) has been synthesized and evaluated for its gene delivery potential [51]. Amphiphilic CS-Lin-Pen displayed molecular self-assembly in aqueous environment to form nontoxic cationic micelles. A simple mixing of CS-Lin-Pen micelles with pDNA solution results in stable polyplexes of CS-Lin-Pen/pDNA due to electrostatic interaction between cationic micelles and anionic pDNA. The resultant polyplexes offered enhanced stability to complexed pDNA against DNase I degradation and facilitated five-fold higher cellular uptake compared to unmodified chitosan. Also, visualization of cellular uptake using confocal microscopy indicates that a large fraction of CS-Lin-Pen/pDNA polyplexes localized to the nuclei, while chitosan/pDNA polyplexes were mainly accumulated around the perinuclear fraction of the cytoplasm. Furthermore, the CS-Lin-Pen/pDNA polyplexes exhibited ∼34–40-fold greater gene transfection than chitosan without modification in three cell lines including HEK 293, CHO, and HeLa. Therefore, micelles of CS-Lin-Pen could be harnessed as a promising nonviral vector for gene therapy applications.

The presence of L-arginine residues is recognized as a critical structural feature of CPPs [133]. Furthermore, it was established that the number of arginine units is also crucial, with oligoarginine of seven to nine residues exhibiting the highest translocation efficiency [134,135]. Considering the membrane translocating ability of oligoarginine, chitosan was chemically conjugated with octaarginine to improve its gene transfection efficiency [128]. The octaarginine functionalized chitosan (R8-CS) was capable of condensing DNA into nanoscale polyplexes of around 100–200 nm size. Conjugation of octa-arginine improved the gene transfection efficiency of chitosan while maintaining low cytotoxicity. Moreover, R8-CS also demonstrated good serum resistance.

### 5.2. CPP Conjugated Chitosan for siRNA Delivery

RNA interference (RNAi) using siRNA has emerged as a potential tool for targeted knockdown of a disease-associated gene [136]. The specific gene knockdown ability of siRNA extends the scope of gene therapy for effective treatment of genetic disorders and cancers. Nevertheless, the widespread clinical application of siRNA is mainly restricted by its ineffective delivery to specific cell populations [137,138,139,140]. However, the recent success in DNA delivery with chitosan-based formulations promotes chitosan as a potential candidate for siRNA delivery. To further improve the cytoplasmic delivery of chitosan-based formulations, several CPPs such as TAT and poly-l-arginine have been chemically conjugated with chitosan. Katas *et al.* [141] developed TAT-peptide conjugated chitosan nanoparticles (CN-TAT) as a potential carrier for siRNA delivery. Chitosan nanoparticles were first formulated by ionic gelation of chitosan with anionic pentasodium tripolyphosphate anions which were subsequently conjugated with TAT-peptide via disulfide linkage. The CN-TAT exhibited good siRNA loading (93% ± 0.01%) and binding efficiency while maintaining high cell viability.

To increase intracellular siRNA delivery, a chitosan-based TAT-peptide conjugated nanoparticle (CS-PEG-TAT) formulation has been developed [130]. Chemical conjugation of TAT-peptide to the chitosan backbone was performed using a heterobifunctional PEG linker. Besides functioning as a linker, PEG diminishes the steric hindrance between cationic chitosan and TAT peptide and improves stability of the CS-PEG-TAT/siRNA polyplex. PEG moiety also builds a protective shield that provides efficient protection to the therapeutic molecules. The conjugation of TAT peptide is known to enhance the siRNA complexation ability of the polymer and also increase the stability of the polyplexes [142]. The cationic CS-PEG-TAT polymer assembled with anionic siGLO Green Transfection Indicator (a scrambled siRNA) to form sterically stable, monodispersed nanoparticles. As expected, grafting of the PEG moiety on the chitosan improved the steric stability that yielded highly monodispersed nanoparticles. At pH 6, the CS-PEG-TAT polymer formed the smallest nanoparticles with a size of 100 nm and demonstrated significantly higher transfection efficiency with minimal toxicity in neuronal cells.

Oligoarginines have been extensively used as a CPP to enhance the intracellular delivery of their cargo. Therefore, conjugation of oligoarginine to chitosan could be a promising approach to enhance the gene silencing efficacy of chitosan/siRNA polyplexes. With this purpose, a nona-arginine functionalized chitosan was synthesized by carbodiimide-mediated coupling reaction between the carboxylate of the peptide and amino groups of chitosan [129]. Conjugation of nona-arginine has been found to improve the siRNA binding affinity of chitosan, which was likely because of the higher surface charge of the nona-arginine conjugated chitosan. The average particle size and surface charge of the nonaarginine-chitosan/siRNA polyplexes are largely dependent on the extent of nona-arginine substitution on chitosan. Both the size and surface charge of the polyplexes were increased with the increasing degree of substitution of nona-arginine. In addition, nonaarginine-chitosan/siRNA polyplexes exhibited enhanced uptake and transfection efficiency in comparison to chitosan/siRNA polyplexes, while preserving high cell viability.

In another study, chitosan was coupled with poly-l-arginine and PEG for efficient siRNA delivery *in vitro* and *in vivo* [127]. The cellular uptake potential of different cationic polymers was measured in Hepa 1–6 cells after complexing with fluorescently labeled dsRNA. Poly-l-arginine conjugated chitosan (CS-PLR) and pegylated CS-PLR (PEG-CS-PLR) exhibited 78% and 79% fluorescence-positive cells, while unmodified chitosan and pegylated chitosan resulted in positive fluorescence in 1.5% and 2.6% cells, respectively. Moreover, the siRNA delivery efficiency of PEG-CS-PLR and CS-PLR was comparable to Lipofectamine 2000. The extent of siRNA-mediated silencing of target genes was highest after treatment of cells using polyarginine conjugated chitosan derivatives such as CS-PLR and PEG-CS-PLR as siRNA carriers. Unlike CS-PLR and Lipofectamine 2000 which have shown decreased siRNA delivery in the presence of a higher proportion of serum (30% and 50%), PEG-CS-PLR demonstrated serum-independent siRNA delivery efficiency. In addition, PEG-CS-PLR displayed higher cell viability and better hemocompatibility as compared to poly-l-arginine. Most importantly, intratumoral administration of PEG-CS-PLR/siRFP resulted in the highest level of RFP protein silencing in tumor tissues compared to all other formulations including CS-PLR/siRFP. Therefore, PEG-CS-PLR could be effective for *in vivo* delivery of therapeutic siRNAs.

## 6. Conclusions and Future Prospects

Chitosan-based nucleic acid delivery is an emerging trend over the last few decades as evidenced by the increasing number of publications and patents. Nevertheless, the clinical success of chitosan-based vectors is averted by their low transfection efficiency and poor cell specificity. The chemical conjugation of CPPs revitalized transfection efficiency of chitosan-based vectors by improving their cellular uptake, while maintaining high levels of cell viability. Therefore, CPP-conjugated chitosan holds great promise in the field of gene therapy. However, the cell specificity still remains unresolved which negatively influences the outcome of the gene therapy. Thus, modification of CPPs sequences or incorporation of artificial amino acids bearing side chains that can increase their specificity is essential and the strategies that would allow CPP-conjugated chitosan to be compatible with systemic administration should facilitate endosomal escape and ensure efficient intracellular delivery. Although CPP-conjugated chitosan polymers are considered relatively safe and nontoxic, long-term *in vivo* toxicity profiling is highly recommended. More importantly, the therapeutic potential of the delivery system should be assessed in a clinically relevant model.
